# Fungal and fungal-like diversity in marine sediments from the maritime Antarctic assessed using DNA metabarcoding

**DOI:** 10.1038/s41598-022-25310-2

**Published:** 2022-12-06

**Authors:** Mayanne Karla da Silva, Láuren Machado Drumond de Souza, Rosemary Vieira, Arthur Ayres Neto, Fabyano A. C. Lopes, Fábio S. de Oliveira, Peter Convey, Micheline Carvalho-Silva, Alysson Wagner Fernandes Duarte, Paulo E. A. S. Câmara, Luiz Henrique Rosa

**Affiliations:** 1 Laboratório de Microbiologia Polar E Conexões Tropicais, Departamento de Microbiologia, Instituto de Ciências Biológicas, Universidade Federal de Minas Gerais, P. O. Box 486, Belo Horizonte, MG 31270-901 Brazil; 2 Instituto de Geociências, Universidade Federal Fluminense, Rio de Janeiro, Brazil; 3 Laboratório de Microbiologia, Universidade Federal Do Tocantins, Porto Nacional, Brazil; 4 Departamento de Geografia, Universidade Federal de Minas, Gerais, Minas Gerais Brazil; 5 British Antarctic Survey, NERC, High Cross, Madingley Road, Cambridge, CB3 0ET UK; 6 Department of Zoology, University of Johannesburg, PO Box 524, Auckland Park, 2006 South Africa; 7 Millennium Institute Biodiversity of Antarctic and Subantarctic Ecosystems (BASE), Las Palmeras 3425, Santiago, Chile; 8 Departamento de Botânica, Universidade de Brasília, Brasília, Brazil; 9 Laboratório de Microbiologia, Imunologia E Parasitologia, Universidade Federal de Alagoas, Arapiraca, Alagoas, Brazil

**Keywords:** Microbiology, Environmental microbiology, Fungi

## Abstract

We assessed the fungal and fungal-like sequence diversity present in marine sediments obtained in the vicinity of the South Shetland Islands (Southern Ocean) using DNA metabarcoding through high-throughput sequencing (HTS). A total of 193,436 DNA reads were detected in sediment obtained from three locations: Walker Bay (Livingston Island) at 52 m depth (48,112 reads), Whalers Bay (Deception Island) at 151 m (104,704) and English Strait at 404 m (40,620). The DNA sequence reads were assigned to 133 distinct fungal amplicon sequence variants (ASVs) representing the phyla *Ascomycota*, *Basidiomycota*, *Mortierellomycota*, *Chytridiomycota*, *Glomeromycota*, *Monoblepharomycota*, *Mucoromycota* and *Rozellomycota* and the fungal-like Straminopila. *Thelebolus balaustiformis*, *Pseudogymnoascus* sp., Fungi sp. 1, *Ciliophora* sp., *Agaricomycetes* sp. and *Chaetoceros* sp. were the dominant assigned taxa. Thirty-eight fungal ASVs could only be assigned to higher taxonomic levels, and may represent taxa not currently included in the available databases or represent new taxa and/or new records for Antarctica. The total fungal community displayed high indices of diversity, richness and moderate to low dominance. However, diversity and taxa distribution varied across the three sampling sites. In Walker Bay, unidentified fungi were dominant in the sequence assemblage. Whalers Bay sediment was dominated by Antarctic endemic and cold-adapted taxa. Sediment from English Strait was dominated by *Ciliophora* sp. and *Chaetoceros* sp. These fungal assemblages were dominated by saprotrophic, plant and animal pathogenic and symbiotic taxa. The detection of an apparently rich and diverse fungal community in these marine sediments reinforces the need for further studies to characterize their richness, functional ecology and potential biotechnological applications.

## Introduction

Although characterized by extreme environmental conditions, Antarctica’s marine ecosystems host biological and particularly microbial communities spanning a gradient of complexity^1^. Their biodiversity, while still imperfectly known, has received research attention. However, microbial diversity, particularly that present in marine benthic ecosystems, remains poorly characterized^2^. Among marine ecosystems, the seafloor represents approximately two-thirds of the Earth’s surface and hosts diverse microbial communities living under extreme conditions imposed by the depth^3,4^. The sediments at the seafloor are recognized as polyextreme ecosystems and display unique characteristics. They face mostly stable conditions, but with an absence of sunlight, low temperatures, high hydrostatic pressure and generally very low nutrient availability^3,5^. They have great potential in the study of the biodiversity, ecology and evolution of different microbial groups.

The Southern Ocean surrounding the Antarctic continent contributes approximately 30% of global ocean area^1^. Southern Ocean ecosystems face a range of challenging extreme environmental conditions^2^, including chronically low temperature, salinity and pH variability and low nutrient availability. Knowledge of the taxonomy and ecology of microorganisms inhabiting different habitats in the Southern Ocean remains in its infancy^6^. These ecosystems host microbiomes including viruses, archaea, bacteria, microalgae and fungi that are practically almost entirely unexplored^6^. Virtually all substrates present in the Southern Ocean, including sediments, rocks, sea ice, seawater, seaweeds, invertebrates and vertebrates are associated with microbial life ^7^.

Fungal communities have been reported from deep-sea sediments of the Atlantic and Pacific Oceans and the South China Sea^7^. However, the application of mycological studies in Southern Ocean ecosystems has to date been very limited^8,9^. The pioneering study of Gonçalves et al.^9^ indicated that Antarctic deep sea sediments provided good potential to recover and study the biology of unknown barophilic/psychrophilic fungi. The application of traditional culturing approaches to Antarctic marine sediments confirmed the presence of members of the fungal phyla *Ascomycota*, *Basidiomycota* and *Mucoromycota*^8,9^. Better characterization of these fungal communities can now be achieved using environmental DNA approaches to assess their complexity, ecological role and biotechnological potential^7^. A recent DNA metabarcoding study also revealed the presence of sequences representing *Chytridiomycota* and *Rozellomycota* in deep-sea sediments from maritime Antarctica^2^. Marine sediments from Antarctica represent unique habitats for the study fungi under polyextreme conditions^9^. In the current study, we documented fungal and fungal-like sequence diversity and distribution in marine sediments obtained from three maritime Antarctic locations in the Southern Ocean in the vicinity of the South Shetland Islands, using DNA metabarcoding through high throughput sequencing (HTS).

## Materials and methods

### Marine sediment sampling

**Figure 1:**
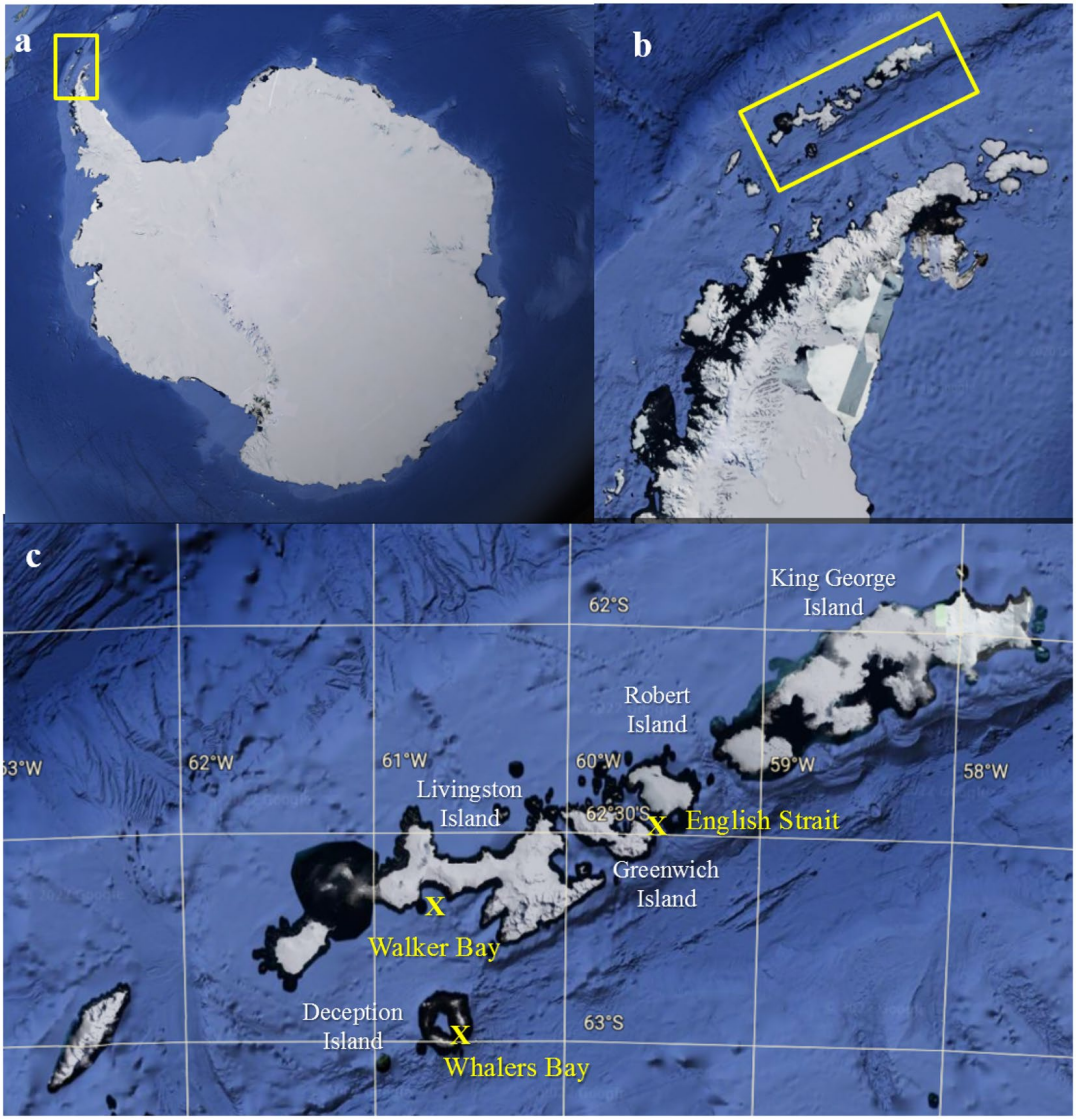
Satellite images of the study region from which samples were obtained, from Google Earth (https://www.google.com.br/maps/) Version 9.174.0.2 based on data from SIO, NOAA and U.S. Navy. (**a**) Antarctica, with the Antarctic Peninsula indicated within the yellow rectangle, (**b**) South Shetland Islands indicated within yellow rectangle, (**c**) sampling locations at Walker Bay (Livingston Island), Whalers Bay (Deception Island) and English Strait. Satellite images **a** and **b** obtained from Google Earth Pro, 2019 (https://earth.google.com).

Marine sediment samples (one per site) were collected from three different locations around the South Shetlands Islands, maritime Antarctica (Fig. 1) during the austral summer in December 2013. Samples were collected using a box corer from Walker Bay (Livingston Island) at 52 m, Whalers Bay (Deception Island) at 151 m and English Strait at 404 m depths. Sections of 10 cm length (approximately 500 g sediment) from the base of each core were selected, sealed, placed in sterile Whirl-pack (Nasco, Ft. Atkinson, WI) bags and frozen at -20 °C until processing in the laboratory at the Federal University of Minas Gerais, Brazil. There, the samples were gradually thawed in sterile conditions at 4 °C for 24 h before carrying out DNA extraction. Three subsamples (5 g) of the central parts of each core were obtained under aseptic conditions and processed, to increase the fungal DNA yield. Thus, nine subsamples (three per core) were used for environmental DNA (eDNA) extraction.

### Physicochemical characteristics of the sediments sampled

A total of 400 g of each sediment sample was used for physicochemical analyses. The granulometric composition of the sediment samples (sand, silt and clay fractions) was assessed in chemically dispersed samples using 10 mL of 1 mol L^-1^ NaOH and stirred slowly for 16 h^10^. The sand fraction (coarse and fine sand) was separated by sieving and the silt and clay fractions by differential sedimentation, using the pipette method. The results were plotted on a ternary sand-silt–clay diagram to identify the textural class.

Chemical analyses of the sediment samples followed the parameters established by Teixeira et al.^10^. Briefly, pH was determined using a 1:2.5 sediment:deionized water ratio. Potential acidity (H + Al) was determinated with 0.5 mol L^−1^ Ca(OAc)_2_ buffered to pH 7 and quantified by titration with 0.0606 mol L^−1^ NaOH. Exchangeable Ca^2+^, Mg^2+^ and Al^3+^ were obtained with 1 mol L^−1^ KCl, and K^+^ and P^+^ with Melich. The element levels in the sediments were determined by ICP (Al^3+^), flame emission (Na^+^, K^+^) and photocolorimetry by the ascorbic acid method (P). Total organic carbon (TOC) was obtained by wet oxidation following the Walkley–Black method. Total cation exchange capacity (CEC) was calculated as the sum of the bases (Ca^2+^, Mg^2+^, K^+^ and Al^3+^) and potential acidity (H^+^  + Al^3+^). All sediment analyses were performed in triplicate.

### DNA extraction, illumina library construction and sequencing

Four replicate sub-samples were taken from each core, from the center of each core section under strict contamination control conditions. Total DNA was extracted from these using the FastDNA Spin Kit for Soil (MPBIO, Ohio, USA), following the manufacturer’s instructions. DNA samples with concentration ≥ 10 ng were selected further analysis. DNA quality was analyzed by agarose gel electrophoresis (1% agarose in 1 × Trisborate-EDTA) and then quantified using the Quanti-iT™ Pico Green dsDNA Assay (Invitrogen). Library construction and DNA amplification were performed following Illumina 16S Metagenomic Sequencing Library Preparation protocol (Part #15,044,223, Rev. B) by Macrogen Inc. (South Korea). The internal transcribed spacer 2 (ITS2) region of the nuclear ribosomal DNA was used as a DNA barcode for molecular species identification of Fungi^11–13^ using the universal primers ITS3 (5’-GCATCGATGAAGAACGCAGC-3’) and ITS4 (5’-TCCTCCGCTTATTGATATGC-3’)^14^. 25-µL reactions were carried out using 2.5 µL of Microbial DNA (5 ng µL^−1^), 5 µL of Amplicon PCR Forward Primer (1 µM), 5 µL of Amplicon PCR Reverse Primer (1 µM), and 12.5 µL of 2 × KAPA HiFi HotStart ReadyMix (Roche, Basel, Switzerland). The amplification PCR consisted of an initial denaturing step at 95 °C for 3 min; 25 amplification cycles at 95 °C for 30 s, 55 °C for 30 s, and 72 °C for 30 s; and a final extension step for 5 min at 72 °C. Index PCRs were performed using the Herculase II Fusion DNA Polymerase Nextera XT Index Kit V2. Quantification of libraries and pooling were performed according to the Illumina instructions. Paired-end sequencing (2 × 300 bp) was performed commercially on a MiSeq platform (Illumina) by Macrogen Inc. (South Korea). All quality controls to avoid contamination of DNA extraction, PCR and sequencing were carried out by Macrogen Inc. In all DNA extraction steps, we proceeded under strict control conditions within a laminar flow hood to recover the fungal DNA and avoid contamination.

### Data analysis and fungal identification

Quality analysis was carried out using BBDuk v. 38.87 in BBmap software^15^ with the following parameters: Illumina adapters removing (Illumina artefacts and the PhiX Control v3 Library); ktrim = l; k = 23; mink = 11; hdist = 1; minlen = 50; tpe; tbo; qtrim = rl; trimq = 20; ftm = 5; maq = 20. The remaining sequences were imported to QIIME2 version 2021.4 (https://qiime2.org/) for bioinformatics analyses^16^. The qiime2-dada2 plugin was used for filtering, dereplication, turn paired-end fastq files into merged, and to remove chimeras, using default parameters^17^. Taxonomic assignments were determined for amplicon sequence variants (ASVs) in three steps. First, ASVs were classified using the qiime2-feature-classifier^18^ classify-sklearn against the UNITE Eukaryotes ITS database version 8.3^19^. Second, remaining unclassified ASVs were filtered and aligned against the filtered NCBI non-redundant nucleotide sequences (nt) database (October 2021) using BLASTn^20^ with default parameters; the nt database was filtered using the following keywords: “ITS1”, “ITS2”, “Internal transcribed spacer” and “internal transcribed spacer”. Third, output files from BLASTn^20^ were imported to MEGAN6^21^ and taxonomic assignments were performed using the “megan-nucl-Jan2021.db” mapping file with default parameters and trained with Naive Bayes classifier and a confidence threshold of 98.5%. Taxonomic profiles were plotted using the Krona^22^. The heatmap of ASV relative abundance and clustering analysis were performed using Heatmapper^23^; clustering analysis was performed using the following parameters: Average Linkage, Spearman Rank Correlation, and Z-score among samples for each ASV.

We recognize that the number of reads obtained are affected by different factors, such as biomass, DNA extraction and PCR protocols, primer bias, multi-copy genes and multicellular species^24^, which can lead to misinterpretation of absolute abundance^25^. However, we followed the conclusion of Giner et al.^26^, who suggested that such biases did not affect the proportionality between reads and cell abundance, in other words that more reads are linked with higher abundance^27,28^. Therefore, for comparative purposes, we used the number of reads as a proxy for relative abundance. We used the fungal taxonomic classifications proposed by Kirk et al.^29^ and Tedersoo et al.^30^, and MycoBank (http://www.mycobank.org) and the Index Fungorum (http://www.indexfungorum.org) database.

### Fungal diversity and ecology

The relative abundances of the ASVs were used to quantify the fungal taxa present in the sediments sampled, where fungal ASVs with relative abundance > 10% were considered dominant, those between 1 and 10% as intermediate and those with < 1% as minor (rare) components of the fungal community^31^. The relative abundances were used to assess taxon diversity, richness and dominance, using the following indices: (i) Fisher’s α, (ii) Margalef’s and (iii) Simpson’s, respectively. In addition, species accumulation curves were obtained using the Mao Tao index (based on a presence-absence matrix). All results were obtained with 95% confidence, and bootstrap values were calculated from 1000 replicates using the PAST computer program 1.90^32^. Functional ecology assignments of fungal ASVs at generic level were prepared using FunGuild following Nguyen et al.^33^, which can be accessed at http://www.funguild.org/.

### Ethics approval

The collections and studies performed were authorized by the PROANTAR.

## Results

### Fungal taxonomy

**Figure 2:**
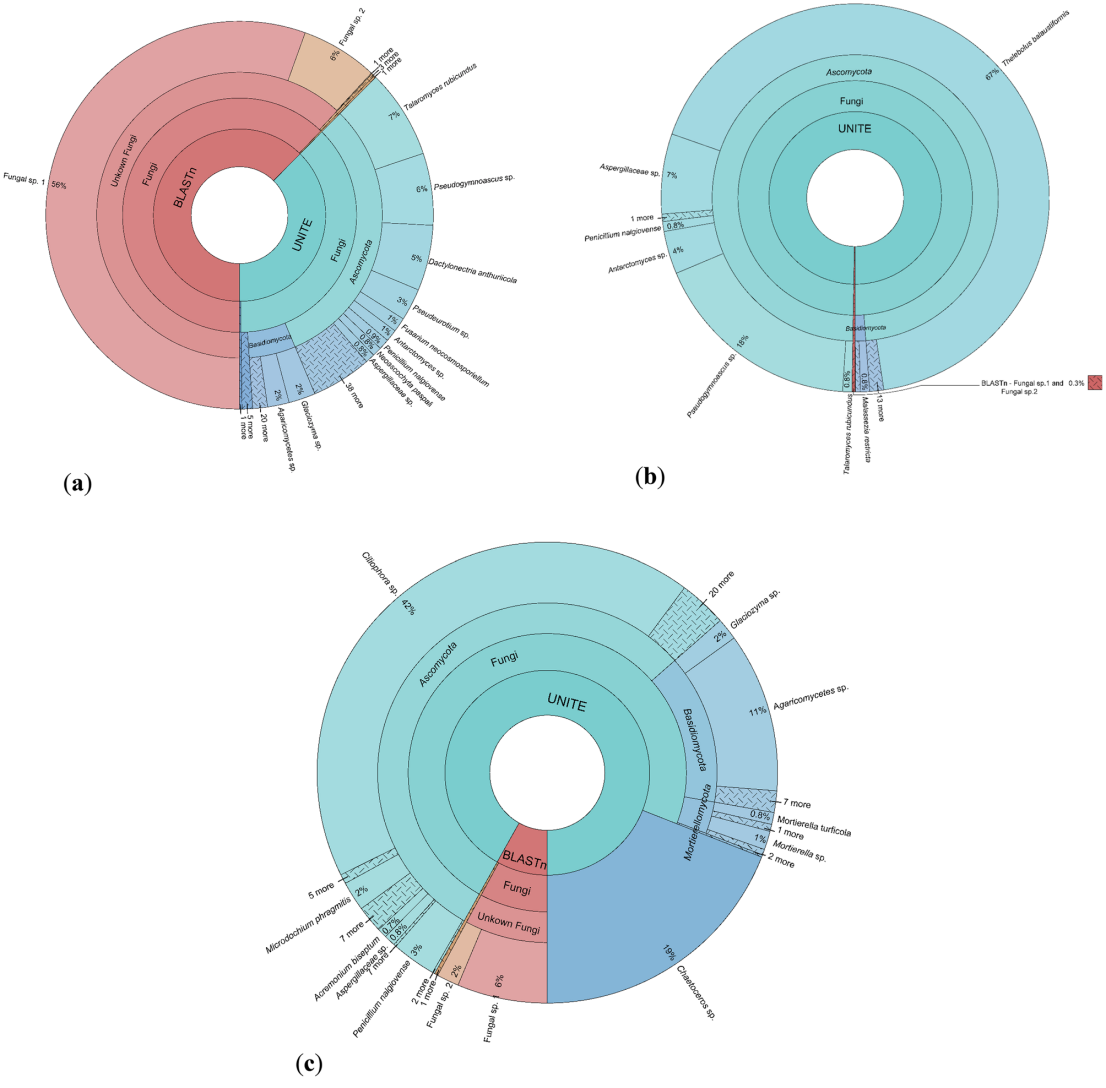
Krona chart showing the abundances of different fungal taxonomic levels detected in marine sediments collected from the South Shetlands Islands, maritime Antarctica. (**a**) Walker Bay (Livingston Island) at 52 m, (**b**) Whalers Bay (Deception Island) at 151 m and (**c**) English Strait at 404 m depths.

A total of 193,436 DNA reads were obtained from the sediments analysed, including 48,112 from Walker Bay (Livingston Island), 104,704 from Whalers Bay (Deception Island) and 40,620 from English Strait. The DNA reads were assigned to 133 distinct fungal ASVs, with 88 recorded from Walker Bay, 32 from Whalers Bay and 59 from English Strait (Supplementary Table S1). The fungal sequence diversity was dominated, in rank order, by taxa representing the phyla *Ascomycota*, *Basidiomycota*, *Mortierellomycota*, *Chytridiomycota*, *Glomeromycota*, *Monoblepharomycota*, *Mucoromycota* and *Rozellomycota*. In addition, the fungal-like Straminopila kingdom was also detected in high abundance. The fungal communities differed in relative abundance profiles between the three sampling locations (Fig. 2 and Supplementary Figure S1). The sequence assemblages detected in the sediment samples from Whalers Bay and English Strait were primarily identified using the UNITE and GenBank databases at taxonomic levels below that of kingdom. In contrast, the majority of the sequence assemblage present in Walker Bay sediment could not be assigned from either database and 55.486% of the ASVs could only be identified as Fungi sp.

Five dominant fungal ASVs were detected, with 16 intermediate ASVs and 110 minority ASVs (Supplementary Table S1). The dominant fungal taxa were *Thelebolus balaustiformis*, *Pseudogymnoascus* sp., Fungi sp. 1, *Ciliophora* sp. and *Agaricomycetes* sp., in rank order. In addition, the fungal-like *Chaetoceros* sp. (Straminopila) was the fourth most dominant taxon assigned. The patterns of dominance among the fungal assemblages of three sampling locations differed. In Walker Bay, Fungi sp. 1 was the dominant ASV (55.486%), while in Whalers Bay *T. balaustiformis* dominated (67.3%) followed by *Pseudogymnoascus* sp. (17.611%) and, in English Strait, *Ciliophora* sp. (42.458%) and *Chaetoceros* sp. (19.031%) dominated the assemblages.

### Fungal diversity in relation to physicochemical properties of marine sediments

The Mao Tao rarefaction indices of the fungal assemblages detected in the sediment samples from the three sites displayed different curves (Supplementary Figure S2). Those from Walker Bay (Livington Island) and Whalers Bay (Deception Island), reached asymptote, suggesting that the data obtained provided a good description of the diversity present. However, the fungal assemblage detected from the English Strait sediment did not reach asymptote, suggesting that the diversity could be greater at this site, which might be revealed by further sampling. The total fungal community detected displayed high indices of diversity (Fisher’s α), richness (Margalef) and moderate to low dominance (Simpson). However, the diversity indices varied across the three sampling sites (Table 1). The highest values of diversity and richness were obtained at Walker Bay, followed by English Strait and Whalers Bay.

**Table 1:** Sampling locations, characteristics, sediment physicochemical data and diversity indices of fungal assemblages detected in the three marine sediments analysed from the South Shetland Islands, maritime Antarctica.

Parameters	Location (depth in m)		
	Walker Bay, Livingston Island (52 m)	Whalers Bay, Deception Island (151 m)	English Strait (404 m)
Location	62°39′149"S; 060°38′225" W	62°56′23"S; 060°39'.42"W	62°28′680″S; 59°32′706″W
**Sediment physical parameters**			
Clay (%)	18	26	15
Silt	43	36	72
Coarse sand	2	1	1
Fine sand	37	43	12
Textural class	Loam	Loam	Silt-loam
**Sediment chemical parameters**			
pH in H_2_O	8.6	8.4	7.8
Exchangeable P—mg dm^–3^	131.8	165.5	135.7
Sum of exchangeable bases Ca + K + Mg (SB) – cmol_c_ dm^-3^	6.74	6.30	6.07
Percentage of base saturation (PBS)—%	100.0	100.0	100.0
H + Al—potential acidity—cmol_c_ dm^-3^	0.0	0.0	0.0
Cation exchange capacity at pH 7 (CEC)—cmol_c_ dm^-3^	6.74	6.30	6.07
Total organic carbon (TOC)—dag kg^-1^	0.77	0.85	0.98
Micronutrient Fe—mg dm^-3^	478.2	617.2	1029.2
Micronutrient Mn—mg dm^-3^	14.9	14.8	43.3
**Fungal diversity indices**			
Number of DNA reads	48,112	104,704	40,620
Number of taxa	89	32	59
Fisher’s-α (diversity)	389	16.3	60.4
Margalef (richness)	19.11	6.73	12.6
Simpson's (dominance)	0.7	0.5	0.8

**Figure 3:**
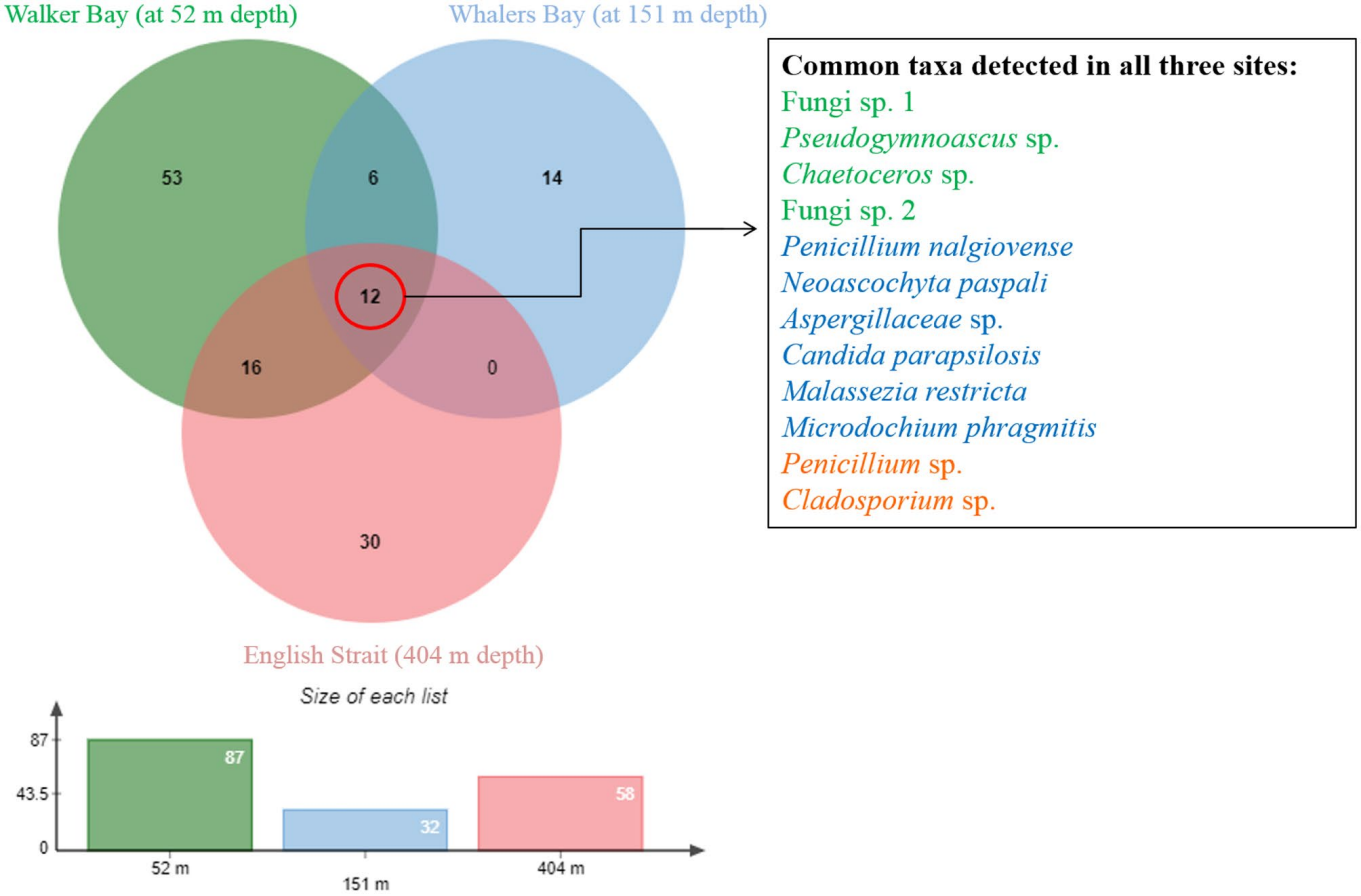
Venn diagram showing the distribution of fungal amplicon sequence variants (ASVs) across the sediment samples obtained at Walker Bay, Whalers Bay and English Strait, South Shetland Islands, maritime Antarctic. In the key, green represents the dominant taxa, blue those of intermediate abundance and orange the rare members of the fungal community.

Of the 133 fungal ASVs assigned, only 12 (9%) were detected at all three sampling locations, with these including taxa from different genera (Fig. 3; Supplementary Table S2). Fungal distribution varied between the three locations, with each hosting some specific fungal taxa. The Walker Bay sediment exclusively hosted 53 ASVs, followed by English Strait with 30 ASVs. The ASVs common to the three locations included abundant (Fungi sp. 1, *Pseudogymnoascus* sp., *Chaetoceros* sp.), intermediate (Fungi sp. 2, *Penicillium nalgiovense*, *Neoascochyta paspali*, *Aspergillaceae* sp., *Candida parapsilosis* and *Microdochium phragmitis*) and minor (*Penicillium* sp. and *Cladosporium* sp.) abundance taxa.

When the physicochemical characteristics were compared with fungal assemblage diversity, Walker Bay and Whalers Bay sediments had the same textural class (loam) and similar granulometric fractions [sand, silt and clay (Table 1)], with the fine sand fraction standing out in relation to the coarse sand fraction. In contrast, English Strait sediment had a higher silt content (silt-loam texture) and was a finer texture in comparison with the other two samples. All three sediments showed alkaline reactions, with high pH values close to or greater than 8. They also showed eutrophic characteristics, with total base saturation values, although the CTC values were not high, always close to 6 cmolc dm^-3^. Organic carbon content was very low (< 1%) and P content was moderate for Antarctic conditions, suggesting all the sediments were influenced by external sources of phosphorus.

### Ecological profile

Functional ecology assignments of the ASVs detected at generic level (Supplementary Table S3 online) indicated that the fungal assemblages present in the three sediment samples were dominated by saprotrophic, plant and animal pathogenic and symbiotic taxa. Further, among the intermediate and minor abundance taxa, a number that are also recognized as important animal pathogens were detected, including *Candida parapsilosis*, *Malassezia restricta* (intermediate), *Aspergillus thermomutatus*, *C. tropicalis*, *Cryptococcus neoformans*, *M. arunalokei*, *M. globosa*, *M. japonica* and *M. sympodialis* (minor).

## Discussion

### Taxonomy and fungal diversity

This study set out to explore the fungal sequence diversity present in marine sediments sampled at three locations in the South Shetland Islands archipelago, maritime Antarctica. In contrast with terrestrial Antarctic environments, to date few studies have attempted to assess fungal diversity in marine sediments in the Southern Ocean^4^. Amongst the studies that are available, most applied traditional cultivation approaches, reporting the presence of relatively low diversity^8,9,34–37^. The application of a DNA metabarcoding approach in the current study detected a richer fungal sequence diversity than indicated by these previous studies.

A small number of studies have focused on the presence of uncultured fungi in Antarctic marine sediments. Lopez-Garcia et al.^38^ used phylogenetic information from ribosomal RNA genes directly amplified from sediment samples to assess the resident biota, but only detected one unidentified fungal taxon in the aphotic zone between 250 and 3000 m depth south of the Antarctic Polar Front. Recently, Ogaki et al.^2^ assessed fungal diversity in deep-sea sediments obtained from different depths in the Southern Ocean using the ITS2 region of nuclear ribosomal DNA using the same metabarcoding methodology as used here. The current study detected a lower fungal sequence diversity than that reported by Ogaki et al.^2^, who detected 655,991 DNA fungal reads and 263 fungal ASVs in sediments sampled at 153, 250, 550 and 1,463 m depth, dominated by *Ascomycota*, *Basidiomycota*, *Mortierellomycota*, *Mucoromycota*, *Chytridiomycota* and *Rozellomycota*. Although only detecting about half of the ASV diversity as reported by Ogaki et al.^2^, we also detected the fungal-like Straminopila. Ogaki et al.^2^ reported the dominant fungal taxa to be representatives of the genera *Mortierella*, *Penicillium*, *Cladosporium*, *Pseudogymnoascus* and *Phaeosphaeria*, from sediment samples collected at different depths to those in the current study. In contrast, in our study the genera *Thelebolus* and *Ciliophora* (fungi), and *Chaetoceros* (Straminopila) were dominant. These different fungal assemblage compositions might relate to the geological characteristics and depths of the sediments sampled. Only seven taxa were shared between the current study and that of Ogaki et al.^2^ (Supplementary Figure S3), which included psychrophilic (*Pseudogymnoascus*), cosmopolitan decomposer (*Cladosporium* and *Penicillium*) and animal opportunistic (*Candida* and *Malassezia*) taxa. The taxa *T. balaustiformis*, Fungi sp. 1, *Ciliophora* sp., *Agaricomycetes* sp. (Fungi) and the fungal-like *Chaetoceros* sp. (Straminopila) dominated the environmental DNA fungal community in terms of relative abundance. *Thelebolus balaustiformis* is a recently described species isolated from tissues of the sponge *Dysidea fragilis* from Gurraig Sound, on the Atlantic coast of Ireland^39^. In Antarctica, *T. balaustiformis* was detected recently in culturing studies of seasonal snow^40^ and glacial ice^41^, without any assessment of ecological functional role. *Ciliophora* is a genus of *Ascomycota* reported to be saprophytic and recovered from tropical environments of Central America^42^, with currently only two known species, *C. cryptica* and *C. quercus* (www.mycobank.org). In Antarctica, sequences assigned to *Ciliophora* have been detected with low and medium relative abundance in DNA metabarcoding studies of lake sediments^43,44^ and soils^45^ from James Ross Island, and in wood samples from historic anthropogenic structures on Deception Island^46^. The fungal-like Straminopila genus *Chaetoceros* was described by C. Ehrenberg in 1844 based on Antarctic material containing the type species *C. dichaeta*, and also occurs in other oceans including in the Arctic^47^. Thirty-eight fungal ASVs could only be assigned to higher taxonomic levels (phylum, class, order, family), and may represent currently unknown taxa, taxa not included in the UNITE or GenBank databases or represent new taxa and/or new records for Antarctica.

### Fungal diversity, distribution and sediment characteristics

All three sediments sampled had similar physicochemical attributes. The general geology of the sampled areas is broadly similar, with rocks of andesitic to basic composition dominating^48^, although of different ages. However, the distribution and diversity of assigned fungal taxa differed considerably between the three locations sampled. Walker Bay, where the ASV Fungi sp. was dominant in the assemblage, is located on Livingston Island, situated between John Beach and Hannah Point (where a number of penguin colonies are located) and receives meltwater from the Verila Glacier. However, none of the physicochemical parameters assessed seem to be related to the dominance of unidentified fungi in the sediment sample from Walker Bay.

Deception Island is a geologically relatively young volcanic island and it has been suggested that aspects of its ecosystems are still at an early stage of development^12^ However, the data reported here from marine sediments obtained within Deception Island’s flooded caldera contrast with those reported by Rosa et al.^12^ from the adjacent Whalers Bay terrestrial soils. Using the same DNA metabarcoding approach, Rosa et al.^12^ detected high dominance of cosmopolitan fungi in the soils, while in the marine sediments we detected a community dominated by *T. balaustiformis* and *Pseudogymnoascus* sp., which are cold-adapted fungi reported from many Antarctic locations. Environmental conditions do differ considerably between the two habitats. Terrestrial habitats of Whalers Bay are locally influenced by geothermal warming, which may influence the diversity of the resident fungal assemblages. In contrast, cold conditions characterise the deeper waters of Whalers Bay. In the English Strait, *Ciliophora* sp. (Fungi) and *Chaetoceros* sp. (Straminopila) dominated the assemblages detected. This location was the deepest sampled (404 m) and displayed the highest concentrations of silt, Fe and Mn, characteristics that might favour the dominance of both taxa.

### Ecological inferences

The ecological roles played by Antarctic marine fungi are generally poorly known. Ecological inferences about the fungal community detected here are similar to those reported by Ogaki et al.^2^ and include taxa recognized as saprophytes, mutualists, symbionts and/or parasites. A majority of the genera assigned are cosmopolitan/decomposer fungi and, if they are metabolically active in this environment, they will likely be involved in the degradation of the limited available organic matter and hence contribute to the local carbon cycle. Among the dominant fungi, the psychrophilic genera *Thelebolus* and *Pseudogymnoascus* and the autotrophic *Chaetoceros* sp. (Straminopila) were detected. In addition, recognized plant and animal pathogens were detected with different abundances, including *Aspergillus*, *Candida*, *Cryptococcus*, *Cutaneotrichosporon*, *Fusarium*, *Malassezia* and *Rhodotorula*. The same functional ecological profile detected for fungi present in marine sediments here have also been reported in studies sampling fungi in soil^12^, air^31^, lake sediment^43^ and rocks^49^ in Antarctica. However, we recognize that, as this study detected environmental DNA, further functional ecological studies are required to better understand the role and importance of fungi in these Antarctic marine sediments. Our data reinforce previous suggestions that Antarctic deep-sea sediments, despite their extreme environmental conditions, may host complex microbial ecosystems^2^.

## Conclusions

Knowledge of the mycobiota present in Antarctic marine sediments of Antarctica has increasing recently, primarily based on the outcomes of traditional culturing studies. To date, few studies in this environment have focused on the detection of fungi through their environmental DNA. Our metabarcoding study confirmed that the three different sediments studied contained a rich and diverse DNA sequence diversity, including both known cosmopolitan and Antarctic endemic taxa with inferred functions as decomposers, plant and animal pathogens, symbionts and, possibly new and undescribed fungal species. The fungal sequence assemblages obtained from the three sediments sampled displayed distinct diversity and dominance patterns. Although DNA metabarcoding does not confirm that living or viable organisms are present in the samples examined, the dominance of unknown taxa assigned in the samples from Livingston Island suggests that the region may harbour considerable currently unknown fungal diversity. Further targeted studies are required to increase knowledge of the richness, functional ecology and potential biotechnological applications of fungi living in Southern Ocean marine environments.

## Supplementary Information

Supplementary Information 1.

Supplementary Information 2.

Supplementary Information 3.

Supplementary Information 4.

Supplementary Information 5.

Supplementary Information 6.

## Data Availability

The datasets generated and/or analyzed during the current study are available in the NCBI repository under the codes SAMN30815283, SAMN30815284, SAMN30815285, SAMN30815286, SAMN30815287, SAMN30815288, SAMN30815289 and SAMN30815290, which can be accessed in https://www.ncbi.nlm.nih.gov/. All sediment samples analyzed in this paper are stored in the Laboratory of Microbiology at Universidade Federal de Minas Gerais.
