# Supplementary material for: Fungal and fungal-like diversity in marine sediments from the maritime Antarctic assessed using DNA metabarcoding
Source: Sci Rep. 2022 Dec 6;12:21044. doi: 10.1038/s41598-022-25310-2 (PMC9726857; doi:10.1038/s41598-022-25310-2)
Supplement: Supplementary file 1 — Supplementary Information 1. [file 41598_2022_25310_MOESM1_ESM.docx]

**Fungal and fungal-like** **diversity in marine sediments from the maritime Antarctic assessed using DNA metabarcoding**

Mayanne Karla da Silva^1^, Láuren Machado Drumond de Souza^1^, Rosemary Vieira^2^, Arthur Ayres Neto^2^, Fabyano A. C. Lopes^3^, Fábio S. de Oliveira^4^, Peter Convey^5,6,7^, Micheline Carvalho-Silva^8^, Alysson Wagner Fernandes Duarte^9^, Paulo E. A. S. Câmara^8^ and Luiz Henrique Rosa^1^*

^1^Departamento de Microbiologia, Universidade Federal de Minas Gerais, Brazil

^2^Instituto de Geociências, Universidade Federal Fluminense, Rio de Janeiro, Brazil

^3^Laboratório de Microbiologia, Universidade Federal do Tocantins, Porto Nacional, Brazil

^4^Departamento de Geografia, Universidade Federal de Minas, Gerais, Minas Gerais, Brazil

^5^British Antarctic Survey, NERC, High Cross, Madingley Road, Cambridge CB3 0ET, United Kingdom

^6^Department of Zoology, University of Johannesburg, PO Box 524, Auckland Park 2006, South Africa

^7^Millennium Institute Biodiversity of Antarctic and Subantarctic Ecosystems (BASE), Las Palmeras 3425, Santiago, Chile

^8^Departamento de Botânica, Universidade de Brasília, Brasília, Brazil

^9^Laboratório de Microbiologia, Imunologia e Parasitologia, Universidade Federal de Alagoas, Arapiraca, Alagoas, Brazil

*Corresponding author

Laboratório de Microbiologia Polar e Conexões Tropicais, Departamento de Microbiologia, Instituto de Ciências Biológicas, Universidade Federal de Minas Gerais, Belo Horizonte, MG, P. O. Box 486, CEP 31270-901. Tel.: +55-31-3409 2749; Fax: +55-31-3409 2730, Brazil. E-mail: lhrosa@icb.ufmg.br


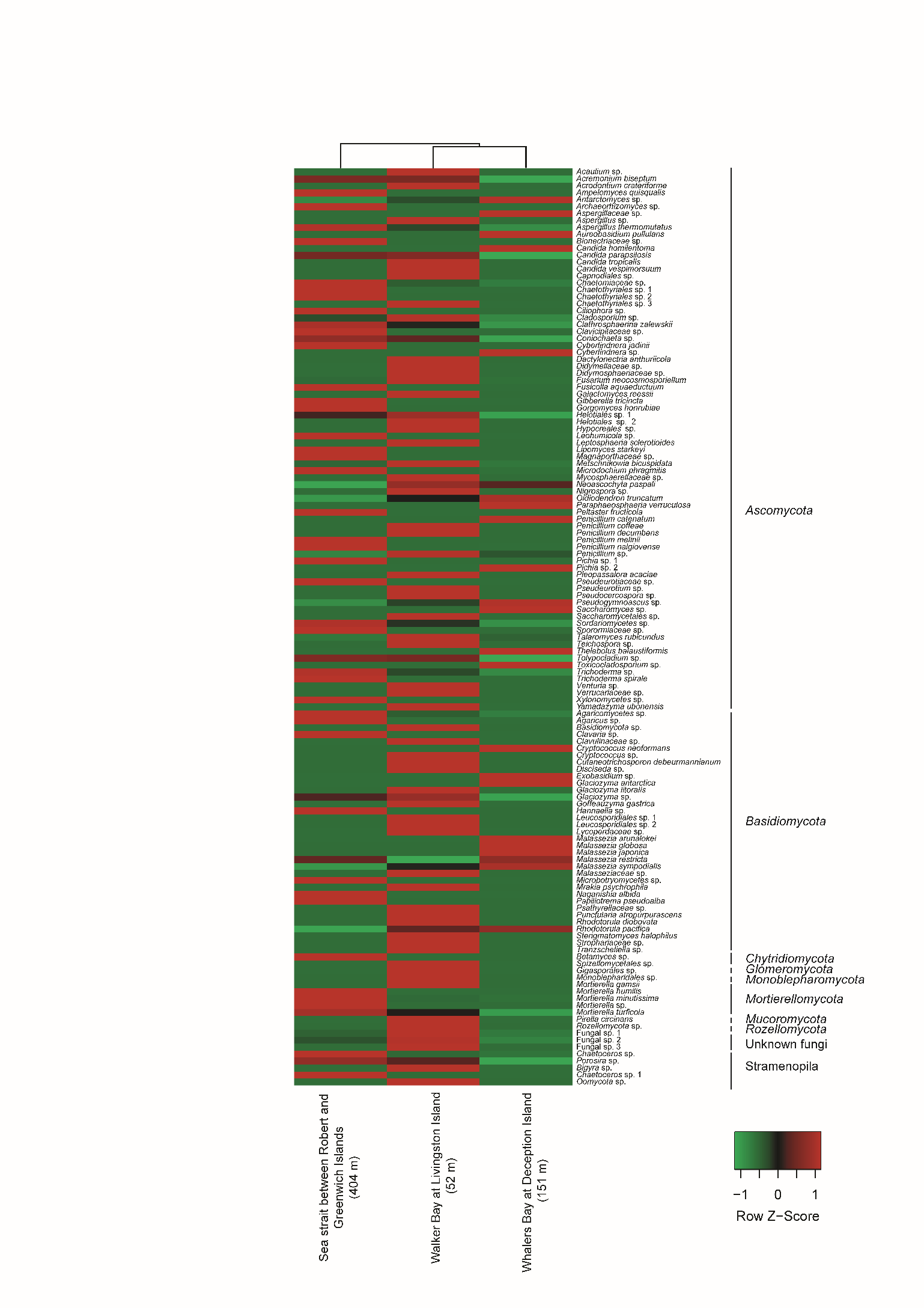


**Supplementary Figure 1**. Heat map of fungal assemblage relative abundances. The color intensities range from red (highest relative abundance) to green (lowest relative abundance). These values represent percentages of DNA fungal reads obtained from sediment samples from Walker Bay (Livingston Island), Whalers Bay (Deception Island) and English Strait. The heatmap of ASV abundance was created using the following parameters: Average Linkage, Spearman Rank Correlation, and Z-score among samples for each ASV.
