# Supplementary material for: Fungal and fungal-like diversity in marine sediments from the maritime Antarctic assessed using DNA metabarcoding
Source: Sci Rep. 2022 Dec 6;12:21044. doi: 10.1038/s41598-022-25310-2 (PMC9726857; doi:10.1038/s41598-022-25310-2)
Supplement: Supplementary file 2 — Supplementary Information 2. [file 41598_2022_25310_MOESM2_ESM.docx]

**Fungal and fungal-like** **diversity in marine sediments from the maritime Antarctic assessed using DNA metabarcoding**

Mayanne Karla da Silva^1^, Láuren Machado Drumond de Souza^1^, Rosemary Vieira^2^, Arthur Ayres Neto^2^, Fabyano A. C. Lopes^3^, Fábio S. de Oliveira^4^, Peter Convey^5,6,7^, Micheline Carvalho-Silva^8^, Alysson Wagner Fernandes Duarte^9^, Paulo E. A. S. Câmara^8^ and Luiz Henrique Rosa^1^*

^1^Departamento de Microbiologia, Universidade Federal de Minas Gerais, Brazil

^2^Instituto de Geociências, Universidade Federal Fluminense, Rio de Janeiro, Brazil

^3^Laboratório de Microbiologia, Universidade Federal do Tocantins, Porto Nacional, Brazil

^4^Departamento de Geografia, Universidade Federal de Minas, Gerais, Minas Gerais, Brazil

^5^British Antarctic Survey, NERC, High Cross, Madingley Road, Cambridge CB3 0ET, United Kingdom

^6^Department of Zoology, University of Johannesburg, PO Box 524, Auckland Park 2006, South Africa

^7^Millennium Institute Biodiversity of Antarctic and Subantarctic Ecosystems (BASE), Las Palmeras 3425, Santiago, Chile

^8^Departamento de Botânica, Universidade de Brasília, Brasília, Brazil

^9^Laboratório de Microbiologia, Imunologia e Parasitologia, Universidade Federal de Alagoas, Arapiraca, Alagoas, Brazil

*Corresponding author

Laboratório de Microbiologia Polar e Conexões Tropicais, Departamento de Microbiologia, Instituto de Ciências Biológicas, Universidade Federal de Minas Gerais, Belo Horizonte, MG, P. O. Box 486, CEP 31270-901. Tel.: +55-31-3409 2749; Fax: +55-31-3409 2730, Brazil. E-mail: lhrosa@icb.ufmg.br


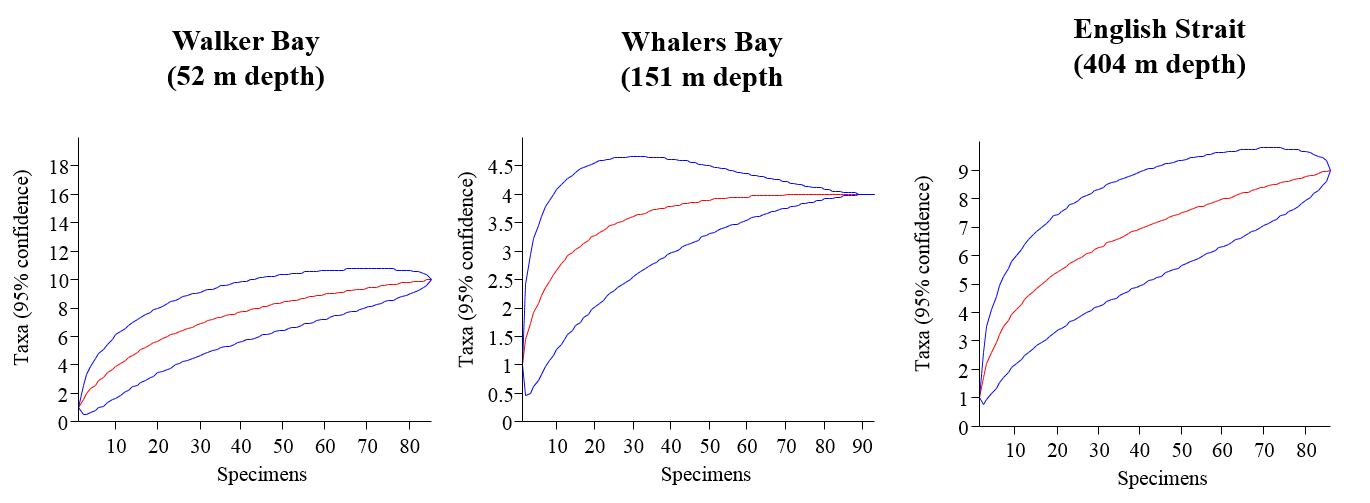


**Supplementary Figure 2**. Rarefaction curves, with 95% confidence limits, of fungal amplicon sequence variants (ASVs) obtained from the three marine sediment samples analysed.
