# Supplementary material for: Fungal and fungal-like diversity in marine sediments from the maritime Antarctic assessed using DNA metabarcoding
Source: Sci Rep. 2022 Dec 6;12:21044. doi: 10.1038/s41598-022-25310-2 (PMC9726857; doi:10.1038/s41598-022-25310-2)
Supplement: Supplementary file 4 — Supplementary Information 4. [file 41598_2022_25310_MOESM4_ESM.docx]

**Fungal and fungal-like** **diversity in marine sediments from the maritime Antarctic assessed using DNA metabarcoding**

Mayanne Karla da Silva^1^, Láuren Machado Drumond de Souza^1^, Rosemary Vieira^2^, Arthur Ayres Neto^2^, Fabyano A. C. Lopes^3^, Fábio S. de Oliveira^4^, Peter Convey^5,6,7^, Micheline Carvalho-Silva^8^, Alysson Wagner Fernandes Duarte^9^, Paulo E. A. S. Câmara^8^ and Luiz Henrique Rosa^1^*

^1^Departamento de Microbiologia, Universidade Federal de Minas Gerais, Brazil

^2^Instituto de Geociências, Universidade Federal Fluminense, Rio de Janeiro, Brazil

^3^Laboratório de Microbiologia, Universidade Federal do Tocantins, Porto Nacional, Brazil

^4^Departamento de Geografia, Universidade Federal de Minas, Gerais, Minas Gerais, Brazil

^5^British Antarctic Survey, NERC, High Cross, Madingley Road, Cambridge CB3 0ET, United Kingdom

^6^Department of Zoology, University of Johannesburg, PO Box 524, Auckland Park 2006, South Africa

^7^Millennium Institute Biodiversity of Antarctic and Subantarctic Ecosystems (BASE), Las Palmeras 3425, Santiago, Chile

^8^Departamento de Botânica, Universidade de Brasília, Brasília, Brazil

^9^Laboratório de Microbiologia, Imunologia e Parasitologia, Universidade Federal de Alagoas, Arapiraca, Alagoas, Brazil

*Corresponding author

Laboratório de Microbiologia Polar e Conexões Tropicais, Departamento de Microbiologia, Instituto de Ciências Biológicas, Universidade Federal de Minas Gerais, Belo Horizonte, MG, P. O. Box 486, CEP 31270-901. Tel.: +55-31-3409 2749; Fax: +55-31-3409 2730, Brazil. E-mail: lhrosa@icb.ufmg.br

**Supplementary Table 1.** Relative abundances of the fungal amplicon sequence variants (ASVs) detected in the three marine sediment sampled from the South Shetland Islands, maritime Antarctica.

|  |  |  |  | **Relative abundance (%)/site (depth)** | | |
| --- | --- | --- | --- | --- | --- | --- |
| **Database** | **Kingdom** | **Phylum** | **Fungal amplicon sequence variant** | **Walker Bay, Livingston Island (52 m)** | **Whalers Bay, Deception Island (151 m)** | **English Strait (404 m)** |
| UNITE | Fungi | *Ascomycota* | *Thelebolus balaustiformis* | 0.602 | 67.301 | 0.000 |
|  |  |  | *Ciliophora* sp. | 0.000 | 0.000 | 42.458 |
|  |  |  | *Pseudogymnoascus* sp. | 5.992 | 17.611 | 0.102 |
|  |  |  | *Aspergillaceae* sp. | 0.783 | 6.671 | 0.795 |
|  |  |  | *Talaromyces rubicundus* | 7.433 | 0.772 | 0.000 |
|  |  |  | *Dactylonectria anthuriicola* | 5.460 | 0.000 | 0.000 |
|  |  |  | *Penicillium nalgiovense* | 0.879 | 0.819 | 3.114 |
|  |  |  | *Antarctomyces* sp. | 1.001 | 3.575 | 0.000 |
|  |  |  | *Pseudeurotium* sp. | 2.683 | 0.000 | 0.000 |
|  |  |  | *Microdochium phragmitis* | 0.110 | 0.007 | 2.067 |
|  |  |  | *Neoascochyta paspali* | 0.784 | 0.621 | 0.191 |
|  |  |  | *Acremonium biseptum* | 0.672 | 0.000 | 0.683 |
|  |  |  | *Candida parapsilosis* | 0.554 | 0.205 | 0.535 |
|  |  |  | *Fusarium neocosmosporiellum* | 1.191 | 0.000 | 0.055 |
|  |  |  | *Penicillium* sp. | 0.485 | 0.265 | 0.201 |
|  |  |  | *Cladosporium* sp. | 0.440 | 0.090 | 0.186 |
|  |  |  | *Trichoderma* sp. | 0.149 | 0.000 | 0.499 |
|  |  |  | *Tolypocladium* sp. | 0.264 | 0.000 | 0.277 |
|  |  |  | *Helotiales* sp. 1 | 0.247 | 0.000 | 0.161 |
|  |  |  | *Trichoderma spirale* | 0.000 | 0.000 | 0.399 |
|  |  |  | *Chaetomiaceae* sp. | 0.047 | 0.000 | 0.321 |
|  |  |  | *Paraphaeosphaeria verruculosa* | 0.000 | 0.278 | 0.000 |
|  |  |  | *Fusicolla aquaeductuum* | 0.000 | 0.000 | 0.272 |
|  |  |  | *Chaetothyriales* sp. 1 | 0.000 | 0.000 | 0.265 |
|  |  |  | *Gibberella tricincta* | 0.000 | 0.000 | 0.249 |
|  |  |  | *Chaetothyriales* sp. 2 | 0.000 | 0.000 | 0.233 |
|  |  |  | *Metschnikowia bicuspidata* | 0.208 | 0.000 | 0.017 |
|  |  |  | *Ampelomyces quisqualis* | 0.000 | 0.000 | 0.220 |
|  |  |  | *Sordariomycetes* sp. | 0.053 | 0.000 | 0.140 |
|  |  |  | *Leohumicola* sp. | 0.000 | 0.000 | 0.193 |
|  |  |  | *Didymosphaeriaceae* sp. | 0.192 | 0.000 | 0.000 |
|  |  |  | *Xylonomycetes* sp. | 0.000 | 0.000 | 0.180 |
|  |  |  | *Lipomyces starkeyi* | 0.000 | 0.000 | 0.177 |
|  |  |  | *Clathrosphaerina zalewskii* | 0.051 | 0.000 | 0.116 |
|  |  |  | *Clavicipitaceae* sp. | 0.000 | 0.000 | 0.145 |
|  |  |  | *Sporormiaceae* sp. | 0.000 | 0.000 | 0.144 |
|  |  |  | *Gorgomyces honrubiae* | 0.000 | 0.000 | 0.143 |
|  |  |  | *Cyberlindnera* sp. | 0.000 | 0.138 | 0.000 |
|  |  |  | *Hypocreales* sp. | 0.134 | 0.000 | 0.000 |
|  |  |  | *Pseudeurotiaceae* sp. | 0.000 | 0.000 | 0.123 |
|  |  |  | *Magnaporthaceae* sp. | 0.000 | 0.000 | 0.119 |
|  |  |  | *Archaeorhizomyces* sp. | 0.000 | 0.000 | 0.110 |
|  |  |  | *Cyberlindnera jadinii* | 0.000 | 0.000 | 0.106 |
|  |  |  | *Peltaster fructicola* | 0.000 | 0.000 | 0.096 |
|  |  |  | *Teichospora* sp. | 0.095 | 0.000 | 0.000 |
|  |  |  | *Bionectriaceae* sp. | 0.000 | 0.000 | 0.082 |
|  |  |  | *Aspergillus thermomutatus* | 0.019 | 0.000 | 0.062 |
|  |  |  | *Didymellaceae* sp. | 0.077 | 0.000 | 0.000 |
|  |  |  | *Helotiales* sp. 2 | 0.067 | 0.000 | 0.000 |
|  |  |  | *Pichia* sp. 1 | 0.000 | 0.000 | 0.064 |
|  |  |  | *Coniochaeta* sp. | 0.024 | 0.000 | 0.031 |
|  |  |  | *Leptosphaeria sclerotioides* | 0.050 | 0.000 | 0.000 |
|  |  |  | *Venturia* sp. | 0.050 | 0.000 | 0.000 |
|  |  |  | *Saccharomyces* sp. | 0.000 | 0.050 | 0.000 |
|  |  |  | *Pleopassalora acaciae* | 0.046 | 0.000 | 0.000 |
|  |  |  | *Candida tropicalis* | 0.043 | 0.000 | 0.000 |
|  |  |  | *Acrodontium crateriforme* | 0.041 | 0.000 | 0.000 |
|  |  |  | *Penicillium melinii* | 0.000 | 0.000 | 0.039 |
|  |  |  | *Candida vespimorsuum* | 0.037 | 0.000 | 0.000 |
|  |  |  | *Yamadazyma ubonensis* | 0.035 | 0.000 | 0.000 |
|  |  |  | *Oidiodendron truncatum* | 0.011 | 0.023 | 0.000 |
|  |  |  | *Aspergillus* sp. | 0.034 | 0.000 | 0.000 |
|  |  |  | *Mycosphaerellaceae* sp. | 0.033 | 0.000 | 0.000 |
|  |  |  | *Candida homilentoma* | 0.000 | 0.033 | 0.000 |
|  |  |  | *Penicillium catenatum* | 0.000 | 0.032 | 0.000 |
|  |  |  | *Penicillium coffeae* | 0.031 | 0.000 | 0.000 |
|  |  |  | *Saccharomycetales* sp. | 0.029 | 0.000 | 0.000 |
|  |  |  | *Aureobasidium pullulans* | 0.000 | 0.028 | 0.000 |
|  |  |  | *Acaulium* sp. | 0.023 | 0.000 | 0.000 |
|  |  |  | *Penicillium decumbens* | 0.018 | 0.000 | 0.000 |
|  |  |  | *Pseudocercospora* sp. | 0.017 | 0.000 | 0.000 |
|  |  |  | *Nigrospora* sp. | 0.016 | 0.000 | 0.000 |
|  |  |  | *Pichia* sp. 2 | 0.000 | 0.014 | 0.000 |
|  |  |  | *Chaetothyriales* sp. 3 | 0.010 | 0.000 | 0.000 |
|  |  |  | *Galactomyces reessii* | 0.008 | 0.000 | 0.000 |
|  |  |  | *Toxicocladosporium* sp. | 0.000 | 0.008 | 0.000 |
|  |  | *Basidiomycota* | *Agaricomycetes* sp. | 1.798 | 0.000 | 11.200 |
|  |  |  | *Glaciozyma* sp. | 2.236 | 0.000 | 1.541 |
|  |  |  | *Malassezia restricta* | 0.216 | 0.756 | 0.647 |
|  |  |  | *Clavaria* sp. | 0.000 | 0.000 | 0.220 |
|  |  |  | *Papiliotrema pseudoalba* | 0.000 | 0.000 | 0.206 |
|  |  |  | *Naganishia albida* | 0.000 | 0.000 | 0.197 |
|  |  |  | *Microbotryomycetes* sp. | 0.000 | 0.000 | 0.156 |
|  |  |  | *Agaricus* sp. | 0.000 | 0.000 | 0.046 |
|  |  |  | *Hannaella* sp. | 0.000 | 0.000 | 0.041 |
|  |  |  | *Clavulinaceae* sp. | 0.023 | 0.000 | 0.000 |
|  |  |  | *Cryptococcus* sp. | 0.023 | 0.000 | 0.000 |
|  |  |  | *Cryptococcus neoformans* | 0.000 | 0.015 | 0.000 |
|  |  |  | *Cutaneotrichosporon debeurmannianum* | 0.016 | 0.000 | 0.000 |
|  |  |  | *Disciseda* sp. | 0.075 | 0.000 | 0.000 |
|  |  |  | *Exobasidium* sp. | 0.000 | 0.007 | 0.000 |
|  |  |  | *Glaciozyma antarctica* | 0.000 | 0.007 | 0.000 |
|  |  |  | *Glaciozyma litoralis* | 0.263 | 0.000 | 0.000 |
|  |  |  | *Goffeauzyma gastrica* | 0.040 | 0.000 | 0.000 |
|  |  |  | *Leucosporidiales* sp. 1 | 0.115 | 0.000 | 0.000 |
|  |  |  | *Leucosporidiales* sp. 2 | 0.016 | 0.000 | 0.000 |
|  |  |  | *Lycoperdaceae* sp. | 0.044 | 0.000 | 0.000 |
|  |  |  | *Malassezia arunalokei* | 0.000 | 0.048 | 0.000 |
|  |  |  | *Malassezia globosa* | 0.000 | 0.193 | 0.000 |
|  |  |  | *Malassezia japonica* | 0.000 | 0.004 | 0.000 |
|  |  |  | *Malassezia sympodialis* | 0.034 | 0.075 | 0.000 |
|  |  |  | *Malasseziaceae* sp. | 0.053 | 0.000 | 0.000 |
|  |  |  | Mrakia psychrophila | 0.009 | 0.000 | 0.000 |
|  |  |  | *Psathyrellaceae* sp. | 0.053 | 0.000 | 0.000 |
|  |  |  | *Punctularia atropurpurascens* | 0.007 | 0.000 | 0.000 |
|  |  |  | *Rhodotorula diobovata* | 0.097 | 0.000 | 0.000 |
|  |  |  | *Rhodotorula pacifica* | 0.056 | 0.071 | 0.000 |
|  |  |  | *Sterigmatomyces halophilus* | 0.039 | 0.000 | 0.000 |
|  |  |  | *Strophariaceae* sp. | 0.029 | 0.000 | 0.000 |
|  |  |  | *Tranzscheliella* sp. | 0.033 | 0.000 | 0.000 |
|  |  | *Mortierellomycota* | *Mortierella* sp. | 0.000 | 0.000 | 1.310 |
|  |  |  | *Mortierella turficola* | 0.451 | 0.000 | 0.833 |
|  |  |  | *Mortierella minutissima* | 0.021 | 0.000 | 0.495 |
|  |  |  | *Mortierella humilis* | 0.000 | 0.000 | 0.393 |
|  |  |  | *Mortierella gamsii* | 0.073 | 0.000 | 0.000 |
|  |  | *Chytridiomycota* | *Betamyces* sp. | 0.000 | 0.000 | 0.171 |
|  |  | *Glomeromycota* | *Gigasporales* sp. | 0.163 | 0.000 | 0.000 |
|  |  | *Monoblepharomycota* | *Monoblepharidales* sp. | 0.090 | 0.000 | 0.000 |
|  |  | *Mucoromycota* | *Pirella circinans* | 0.014 | 0.000 | 0.000 |
|  |  | *Rozellomycota* | *Rozellomycota* sp. | 0.067 | 0.000 | 0.000 |
|  | Stramenopila | *Ochrophyta* | *Chaetoceros* sp. | 0.206 | 0.012 | 19.031 |
| GENBANK | Fungi | *Ascomycota* | *Capnodiales* sp. | 0.051 | 0.000 | 0.000 |
|  |  |  | *Verrucariaceae* sp. | 0.041 | 0.000 | 0.000 |
|  |  | *Basidiomycota* | *Basidiomycota* sp. | 0.135 | 0.000 | 0.000 |
|  |  | *Chytridiomycota* | *Spizellomycetales* sp. | 0.014 | 0.000 | 0.000 |
|  |  | Unidentified | Fungi sp. 1 | 55.489 | 0.164 | 6.339 |
|  |  |  | Fungi sp. 2 | 6.406 | 0.106 | 1.555 |
|  |  |  | Fungi sp. 3 | 0.069 | 0.000 | 0.000 |
|  | Stramenopila | *Oomycota* | *Oomycota* sp. | 0.034 | 0.000 | 0.000 |
|  |  | *Bacillariophyta* | *Porosira* sp. | 0.143 | 0.000 | 0.193 |
|  |  |  | *Chaetoceros* sp. | 0.003 | 0.000 | 0.057 |
|  |  | *Bigyra* | *Bigyra* sp. | 0.004 | 0.000 | 0.000 |

Green indicates dominant, blue intermediate and orange minor relative abundance (see Methods).
