# Supplementary material for: Fungal and fungal-like diversity in marine sediments from the maritime Antarctic assessed using DNA metabarcoding
Source: Sci Rep. 2022 Dec 6;12:21044. doi: 10.1038/s41598-022-25310-2 (PMC9726857; doi:10.1038/s41598-022-25310-2)
Supplement: Supplementary file 6 — Supplementary Information 6. [file 41598_2022_25310_MOESM6_ESM.docx]

**Fungal and fungal-like** **diversity in marine sediments from the maritime Antarctic assessed using DNA metabarcoding**

Mayanne Karla da Silva^1^, Láuren Machado Drumond de Souza^1^, Rosemary Vieira^2^, Arthur Ayres Neto^2^, Fabyano A. C. Lopes^3^, Fábio S. de Oliveira^4^, Peter Convey^5,6,7^, Micheline Carvalho-Silva^8^, Alysson Wagner Fernandes Duarte^9^, Paulo E. A. S. Câmara^8^ and Luiz Henrique Rosa^1^*

^1^Departamento de Microbiologia, Universidade Federal de Minas Gerais, Brazil

^2^Instituto de Geociências, Universidade Federal Fluminense, Rio de Janeiro, Brazil

^3^Laboratório de Microbiologia, Universidade Federal do Tocantins, Porto Nacional, Brazil

^4^Departamento de Geografia, Universidade Federal de Minas, Gerais, Minas Gerais, Brazil

^5^British Antarctic Survey, NERC, High Cross, Madingley Road, Cambridge CB3 0ET, United Kingdom

^6^Department of Zoology, University of Johannesburg, PO Box 524, Auckland Park 2006, South Africa

^7^Millennium Institute Biodiversity of Antarctic and Subantarctic Ecosystems (BASE), Las Palmeras 3425, Santiago, Chile

^8^Departamento de Botânica, Universidade de Brasília, Brasília, Brazil

^9^Laboratório de Microbiologia, Imunologia e Parasitologia, Universidade Federal de Alagoas, Arapiraca, Alagoas, Brazil

*Corresponding author

Laboratório de Microbiologia Polar e Conexões Tropicais, Departamento de Microbiologia, Instituto de Ciências Biológicas, Universidade Federal de Minas Gerais, Belo Horizonte, MG, P. O. Box 486, CEP 31270-901. Tel.: +55-31-3409 2749; Fax: +55-31-3409 2730, Brazil. E-mail: lhrosa@icb.ufmg.br

**Supplementary Table 3.** Ecological profiles obtained from the FUNGuild database and specific citations at generic level of the fungi detected in marine sediment samples obtained from Walker Bay (at 52 m depth), Whaler Bay (151 m depth) and sea strait between Robert and Greenwich Islands (404 m depth).

| **Genus** | **Walker Bay (52 m)** | **Whaler Bay (151 m)** | **English Strait (404 m)** | **Trophic mode** | **FUNGuild^29^*** |
| --- | --- | --- | --- | --- | --- |
| *Acaulium* | 0.023 | 0.000 | 0.000 | Saprotrophic | Saprotrophic^*^ |
| *Acremonium* | 0.672 | 0.000 | 0.683 | Pathogenic-Saprotrophic-Symbiotic | Animal Pathogen- Endophyte-Fungal-Parasite-Plant Pathogen-Wood Saprotrophic^*^ |
| *Acrodontium* | 0.041 | 0.000 | 0.000 | Pathogenic | Plant Pathogen^*^ |
| *Agaricus* | 0.000 | 0.000 | 0.046 | Saprotrophic | Saprotrophic (Kirk et al. 2011) |
| *Ampelomyces* | 0.000 | 0.000 | 0.220 | Saprotrophic-  Symbiotic | Endophyte-Undefined Saprotrophic^*^ |
| *Antarctomyces* | 1.001 | 3.575 | 0.000 | Saprotrophic | Undefined Saprotrophic^*^ |
| *Archaeorhizomyces* | 0.000 | 0.000 | 0.110 | Saprotrophic | Soil Saprotrophic (Menkis et al. 2014) |
| *Aspergillus* | 0.026 | 0.000 | 0.031 | Pathogenic-Saprotrophic | Animal Pathogen-Undefined Saprotrophic^*^ |
| *Aureobasidium* | 0.000 | 0.028 | 0.000 | Pathogenic-Saprotrophic-Symbiotic | Animal Pathogen-Endophyte-Epiphyte-Plant Pathogen-Undefined Saprotroph^*^ |
| *Betamyces* | 0.000 | 0.000 | 0.171 | Pathogenic-Saprotrophic | Plant Pathogen^*^ |
| *Candida* | 0.158 | 0.059 | 0.133 | Pathogenic-Saprotrophic-Symbiotic | Animal Pathogen-Endophyte-Endosymbiont-Epiphyte-Soil Saprotroph^*^ |
| *Chaetoceros* | 0.206 | 0.012 | 19.031 | Autotrophic | Photosynthetic-Phytoplankton (Not et al. 2012) |
| *Ciliophora* | 0.000 | 0.000 | 42.458 | Symbiotic | Endophyte (Sun & Guo 2007) |
| *Cladosporium* | 0.440 | 0.090 | 0.186 | Symbiotic | Endophyte^*^ |
| *Clathrosphaerina* | 0.051 | 0.000 | 0.116 | Saprotrophic | Undefined Saprotrophic^*^ |
| *Clavaria* | 0.000 | 0.000 | 0.220 | Saprotrophic | Undefined Saprotrophic^*^ |
| *Coniochaeta* | 0.024 | 0.000 | 0.031 | Pathogenic-Saprotrophic-Symbiotic | Animal Pathogen-Dung Saprotrophic-Endophyte-Lichen Parasite-Plant Pathogen-Undefined Saprotrophic^*^ |
| *Cryptococcus* | 0.011 | 0.007 | 0.000 | Pathogenic-Saprotrophic-Symbiotic | Animal Pathogen-Endophyte-Epiphyte-Undefined Saprotroph* |
| *Cutaneotrichosporon* | 0.016 | 0.000 | 0.000 | Pathogenic-Saprotrophic | Animal Pathogen^*^- Undefined Saprotrophic (Li et al. 2020) |
| *Cyberlindnera* | 0.000 | 0.069 | 0.053 | Symbiotic | Insect Symbiont (Soto-Robes et al. 2019) |
| *Dactylonectria* | 5.460 | 0.000 | 0.000 | Pathogenic-Saprotrophic | Soil Saprotrophic - Plant Pathogen (Gramaje et al. 2020) |
| *Disciseda* | 0.075 | 0.000 | 0.000 | Saprotrophic | Soil Saprotrophic* |
| *Exobasidium* | 0.000 | 0.007 | 0.000 | Pathogenic | Plant Pathogen* |
| *Fusarium* | 1.191 | 0.000 | 0.055 | Pathogenic-Saprotrophic-Symbiotic | Animal Pathogen-Endophyte-Lichen-Parasite-Plant Pathogen-Soil Saprotrophic-Wood Saprotrophic* |
| *Fusicolla* | 0.000 | 0.000 | 0.272 | Pathogenic-Symbiotic | Plant Pathogen- Insect Symbiont (Biedermann et al. 2013; Lay et al. 2018) |
| *Galactomyces* | 0.008 | 0.000 | 0.000 | Pathogenic | Plant Pathogen* |
| *Gibberella* | 0.000 | 0.000 | 0.249 | Pathogenic | Plant Pathogen* |
| *Glaciozyma* | 0.833 | 0.002 | 0.513 | Saprotrophic | Undefined Saprotrophic (Mohammadi et al. 2022) |
| *Goffeauzyma* | 0.040 | 0.000 | 0.000 | Saprotrophic | Soil-Acid rock drainage-Acidic water (Liu et al. 2015) |
| *Gorgomyces* | 0.000 | 0.000 | 0.143 | Pathogenic | Nematophagous (Kirk et al. 2011) |
| *Hannaella* | 0.000 | 0.000 | 0.041 | Symbiotic | Epiphyte-Endophyte-Phyllosphere Plant (Gonzaga et al. 2015; Masenya et al. 2021) |
| *Leohumicola* | 0.000 | 0.000 | 0.193 | Saprotrophic | Undefined Saprotrophic* |
| *Leptosphaeria* | 0.050 | 0.000 | 0.000 | Pathogenic | Plant Pathogen* |
| *Lipomyces* | 0.000 | 0.000 | 0.177 | Saprotrophic | Soil Saprotrophic-Insect frass (Yurkov, 2018); Naranjo‐Ortiz & Gabaldón 2019) |
| *Malassezia* | 0.050 | 0.215 | 0.129 | Pathogenic-Saprotrophic | Animal Pathogen-Undefined Saprotrophic* |
| *Metschnikowia* | 0.208 | 0.000 | 0.017 | Pathogenic-Symbiotic | Animal/Plant Pathogen-Endosymbiont (Lachance, 2016; Bao et al. 2021) |
| *Microdochium* | 0.110 | 0.007 | 2.067 | Pathogenic-Symbiotic | Endophyte-Plant Pathogen* |
| *Mortierella* | 0.109 | 0.000 | 0.606 | Saprotrophic-Symbiotic | Endophyte-Litter Saprotrophic-Soil Saprotrophic-Undefined Saprotrophic* |
| *Mrakia* | 0.009 | 0.000 | 0.000 | Saprotrophic | Soil Saprotrophic-Undefined Saprotrophic* |
| *Naganishia* | 0.000 | 0.000 | 0.197 | Saprotrophic | Soil-Flowers (Schmidt, 2017; Zhou et al. 2020) |
| *Neoascochyta* | 0.784 | 0.621 | 0.191 | Pathogenic-Saprotrophic | Animal/Plant Pathogen (Golzar et al. 2019; McGorum et al. 2021) |
| *Nigrospora* | 0.016 | 0.000 | 0.000 | Saprotrophic | Undefined Saprotrophic* |
| *Oidiodendron* | 0.011 | 0.023 | 0.000 | Pathogenic-Symbiotic | Ericoid Mycorrhizal* |
| *Papiliotrema* | 0.000 | 0.000 | 0.206 | Pathogenic | Plant Pathogen (Masenya et al. 2021) |
| *Paraphaeosphaeria* | 0.000 | 0.278 | 0.000 | Saprotrophic | Undefined Saprotrophic* |
| *Peltaster* | 0.000 | 0.000 | 0.096 | Pathogenic | Plant Pathogen* |
| *Penicillium* | 0.235 | 0.186 | 0.559 | Saprotrophic | Dung Saprotrophic-Undefined* Saprotrophic-Wood Saprotrophic* |
| *Pichia* | 0.000 | 0.007 | 0.032 | Pathogenic-Saprotrophic-Symbiotic | Animal-Plant Pathogen- Animal Endosymbiont-Undefined Saprotrophic* |
| *Pirella* | 0.014 | 0.000 | 0.000 | Saprotrophic | Undefined Saprotrophic* |
| *Pleopassalora* | 0.046 | 0.000 | 0.000 | Pathogenic | Plant Pathogen (Beilharz et al. 2004) |
| *Porosira* | 0.143 | 0.000 | 0.193 | Autotrophic | Photosynthetic-Phytoplankton (Not et al. 2012) |
| *Pseudocercospora* | 0.017 | 0.000 | 0.000 | Pathogenic | Plant pathogen* |
| *Pseudeurotium* | 2.683 | 0.000 | 0.000 | Saprotrophic | Undefined Saprotrophic* |
| *Pseudogymnoascus* | 5.992 | 17.611 | 0.102 | Pathogenic-Saprotrophic-Symbiotic | Animal pathogen-Soil Saprotrophic* |
| *Punctularia* | 0.007 | 0.000 | 0.000 | Saprotrophic | Undefined Saprotrophic* |
| *Rhodotorula* | 0.076 | 0.035 | 0.000 | Pathogenic-Saprotrophic | Animal Endosymbiont-Animal Pathogen-Endophyte-Plant Pathogen-Undefined Saprotroph* |
| *Saccharomyces* | 0.000 | 0.050 | 0.000 | Saprotrophic | Undefined Saprotrophic* |
| *Sterigmatomyces* | 0.039 | 0.000 | 0.000 | Pathogenic-Symbiotic | Marine animals Pathogen/Parasite-Insect Symbiont (Zhang et al. 2014; Ali et al. 2017) |
| *Talaromyces* | 7.433 | 0.772 | 0.000 | Saprotrophic | Undefined Saprotrophic* |
| *Teichospora* | 0.095 | 0.000 | 0.000 | Saprotrophic | Undefined Saprotrophic* |
| *Thelebolus* | 0.602 | 67.301 | 0.000 | Saprotrophic-Symbiotic | Dung Saprotrophic-Endophyte-Undefined Saprotrophic* |
| *Tolypocladium* | 0.264 | 0.000 | 0.277 | Pathogenic-Symbiotic | Animal Pathogen-Clavicipitaceous Endophyte-Fungal Parasite* |
| *Toxicocladosporium* | 0.000 | 0.008 | 0.000 | Pathogenic-Symbiotic | Endophyte-Plant Pathogen* |
| *Tranzscheliella* | 0.033 | 0.000 | 0.000 | Pathogenic | Plant Pathogen* |
| *Trichoderma* | 0.074 | 0.000 | 0.449 | Pathogenic-Saprotrophic-Symbiotic | Animal Pathogen-Endophyte-Epiphyte-Fungal Parasite-Plant Pathogen-Wood Saprotrophic* |
| *Venturia* | 0.050 | 0.000 | 0.000 | Pathogenic | Plant Pathogen (González-Domínguez et al. 2017) |
| *Yamadazyma* | 0.035 | 0.000 | 0.000 | Saprotrophic-Symbiotic | Saprotrophic-Endophytic-Animal Symbiont (Yao et al. 2019; Chakraborty et al. 2020; Gao et al. 2021; Valderrama et al. 2021) |

*Functional ecology assignments of fungal amplicon sequence variant at generic levels were prepared using FunGuild proposed by Nguyen et al.^29^.

**References**

Ali, S.S. et al. Screening and characterizing of xylanolytic and xylose-fermenting yeasts isolated from the wood-feeding termite, *Reticulitermes chinensis*. *PLoS One* **12**, e0181141 (2017).

Bao, J. et al. First description of milky disease in the Chinese mitten crab *Eriocheir sinensis* caused by the yeast *Metschnikowia bicuspidata*. *Aquaculture* ***532***, 735984 (2021).

Beilharz, V.C. et al. *Passalora perplexa*, an important pleoanamorphic leaf blight pathogen of *Acacia crassicarpa* in Australia and Indonesia. *Stud. Mycol.* **50**, 471-479 (2004).

Biedermann, P.H. et al. Abundance and dynamics of filamentous fungi in the complex ambrosia gardens of the primitively eusocial beetle *Xyleborinus saxesenii* Ratzeburg (Coleoptera: Curculionidae, Scolytinae). *FEMS Microbiol. Ecol.* **83**, 711-723 (2013).

Chakraborty, A. et al. Core mycobiome and their ecological relevance in the gut of five Ips bark beetles (Coleoptera: Curculionidae: Scolytinae). *Front. Microbiol.* **11**, 568853 (2020).

Gao, W.L. et al. New species of *Yamadazyma* from rotting wood in China. *MycoKeys* **83**, 69 (2021).

Golzar, H. et al. *Neoascochyta* species cause leaf scorch on wheat in Australia. *Austral. Plant Dis. Notes* 14, 1-5 (2019).

Gonzaga, L.L. et al. Endophytic fungi from the genus *Colletotrichum* are abundant in the *Phaseolus vulgaris* and have high genetic diversity. *J. Appl. Microbiol.* **118**, 485-496 (2015).

González-Domínguez, E., Armengol, J., Rossi, V. Biology and epidemiology of *Venturia* species affecting fruit crops: a review. *Front. Plant Sci.* **8**, 1496 (2017).

Gramaje, D. et al. Comparative genomic analysis of *Dactylonectria torresensis* strains from grapevine, soil and weed highlights potential mechanisms in pathogenicity and endophytic lifestyle. *J. Fungi* **6**, 255 (2020).

Kirk, P.M. et al. Dictionary of the Fungi: Co-published by Commonwealth Scientific and Industrial Research Organization (CSIRO). *CABI Pub* (2011).

Lachance, M.A. *Metschnikowia*: half tetrads, a regicide and the fountain of youth. *Yeast* **33**, 563-574 (2016).

Lay, C.Y. et al. Canola root–associated microbiomes in the Canadian prairies. *Front. Microbiol.* **9**, 1188 (2018).

Li, A.H. et al. Diversity and phylogeny of basidiomycetous yeasts from plant leaves and soil: Proposal of two new orders, three new families, eight new genera and one hundred and seven new species. *Stud. Mycol.* **96**, 17-140 (2020).

Liu, X.Z. et al. Towards an integrated phylogenetic classification of the *Tremellomycetes*. *Stud. Mycol.* **81**, 85-147 (2015).

Masenya, K. et al. Pathogen infection influences a distinct microbial community composition in sorghum RILs. *Plant Soi*  **463**, 555–572 (2021).

McGorum, B.C. et al. Equine grass sickness (a multiple systems neuropathy) is associated with alterations in the gastrointestinal mycobiome. *Anim Microbiome* **3**, 70 (2021).

Mohammadi, S. et al. The Cold-Active Endo-β-1,3(4)-Glucanase from a marine psychrophilic yeast, *Glaciozyma antarctica* PI12: heterologous expression, biochemical characterisation, and molecular modeling. *Int. J. Appl. Biol. Pharm. Technol.* **12**, 279–300 (2021).

Menkis, A., Urbina, H., James, T.Y., Rosling, A. *Archaeorhizomyces borealis* sp. nov. and a sequence-based classification of related soil fungal species. *Fungal Biol.* **118**, 943-955 (2014).

Naranjo‐Ortiz, M. A., Gabaldón, T. Fungal evolution: diversity, taxonomy and phylogeny of the Fungi. *Biol. Rev.* **94**, 2101-2137 (2019).

Not, F. et al. Diversity and ecology of eukaryotic marine phytoplankton. In *Advances in Botanical Research* (Vol. 64, pp. 1-53). Academic Press (2012).

Schmidt, S.K. A *Naganishia* in high places: functioning populations or dormant cells from the atmosphere? *Mycology* **8**, 153-163 (2017).

Soto-Robles, L.V. et al. An overview of genes from *Cyberlindnera americana*, a symbiont yeast isolated from the gut of the bark beetle *Dendroctonus rhizophagus* (*Curculionidae*: *Scolytinae*), involved in the detoxification process using genome and transcriptome data. *Front. Microbiol.* **10**, 2180 (2019).

Sun, X., Guo, L.D. Endophytic fungi VI. *Ciliophora quercus* sp. nov. from China. *Nova Hedwigia* **85**, 403-406 (2007).

Valderrama B., et al. Cultivable yeast microbiota from the marine fish species *Genypterus chilensis* and *Seriolella violacea*. J. Fungi **7**, 515 (2021)

Yao, H. et al. Phyllosphere epiphytic and endophytic fungal community and network structures differ in a tropical mangrove ecosystem. *Microbiome* **7**, 57 (2019).

Yurkov, A. M. Yeasts of the soil–obscure but precious. *Yeas* **35**, 369-378 (2018).

Zhang, X.Y. et al. Insights into deep-sea sediment fungal communities from the East Indian Ocean using targeted environmental sequencing combined with traditional cultivation. *PLoS One* **9**, e109118 (2014).

Zhou, Y. et al. *Naganishia floricola* sp. nov., a novel basidiomycetous yeast species isolated from flowers of *Sorbaria sorbifolia*. *Inter. J. Syst. Evol. Microbiol.* **70**, 4496-4501 (2020).
